# A Mechanistic Niche Model for Measuring Species' Distributional Responses to Seasonal Temperature Gradients

**DOI:** 10.1371/journal.pone.0007921

**Published:** 2009-11-20

**Authors:** William B. Monahan

**Affiliations:** Audubon California, Emeryville, California, United States of America; University of Zurich, Switzerland

## Abstract

Niche theory is central to understanding how species respond geographically to climate change. It defines a species' realized niche in a biological community, its fundamental niche as determined by physiology, and its potential niche—the fundamental niche in a given environment or geographic space. However, most predictions of the effects of climate change on species' distributions are limited to correlative models of the realized niche, which assume that species are in distributional equilibrium with respect to the variables or gradients included in the model. Here, I present a mechanistic niche model that measures species' responses to major seasonal temperature gradients that interact with the physiology of the organism. I then use lethal physiological temperatures to parameterize the model for bird species in North and South America and show that most focal bird species are not in direct physiological equilibrium with the gradients. Results also show that most focal bird species possess broad thermal tolerances encompassing novel climates that could become available with climate change. I conclude with discussion of how mechanistic niche models may be used to (i) gain insights into the processes that cause species to respond to climate change and (ii) build more accurate correlative distribution models in birds and other species.

## Introduction

Correlative niche models are commonly used to predict species' geographic responses to climate change [1]. These models assume that species' distributions are proximately shaped by major climate variables, either directly through physiological limits or indirectly through other environmental factors that are influenced by climate [2], [3]. When projected beyond the set of climatic conditions used to train the model, correlative niche models further assume that the physiological limits and indirect climatic influences remain relatively constant over space and time [4], [5]. Mounting evidence suggests that many native species conform to these assumptions over a wide range of spatiotemporal scales [6]–[10]. However, we still do not understand precisely how most species' geographic distributions are governed by climate [11]–[13]. Here, I use lethal physiological temperatures to develop a generalized mechanistic niche model that evaluates species' responses to seasonal temperature gradients.

Joseph Grinnell was the first to consider the role of the niche in limiting species' distributions [14]. While Grinnell focused primarily on temperature and its interactions with the physiological limits of the organism, he recognized that species' distributions were further shaped within these constraints by other factors such as relative humidity, physical barriers to dispersal, resource availability, and biotic interactions [15]. From a mechanistic perspective, understanding species' complex direct and indirect responses to climate change requires that we first understand in the simplest possible sense how their physiological limits relate to temperature and whether they are realized in geographic space. This knowledge is critical for forecasting the potential future movements of species because climate change is expected to generate novel climates [16] and species are capable of unexpectedly colonizing new environments [17], [18]. In brief, we need a simple model that clearly delineates where in environmental space a species could conceivably exist and, by extension, where responses to climate change are necessarily undefined.

An important framework for developing such a model was advanced by Hutchinson [19], who distinguished the multidimensional environmental space where a species could exist (fundamental niche) from the subset of this space where the species actually coexists in a community (realized niche). The fundamental niche is traditionally regarded as an area of environmental space where the per capita growth rate of a population, or its mean absolute fitness, is greater than or equal to one [20], [21]. Importantly, the size, shape, and position of a species' fundamental niche may change through time as a consequence of adaptive, plastic, demographic, and stochastic processes operating on the underlying suite of organismal traits [22]–[24]. Furthermore, these processes can occur both frequently and rapidly near the margins of a species' distribution in geographic or environmental space [25]–[28]. Taken in combination, species are anticipated to respond to ongoing changes in climate in the extreme or extralimital areas of a distribution, and it is thus important to consider potential movements in population sinks that lie beyond a presently defined niche [21], [28].

Species' potential movements also need to be considered in relation to the climates that are available. Importantly, not all climates exist in the geographic domain at a given point in time, only those defined by the realized climate space [16]. By extension, not all portions of a species' fundamental niche are necessarily represented in the geographic domain; the portions that are represented – as defined by the intersection of the fundamental niche with a realized climate space – comprise what is termed the potential niche [29]. Understanding how species' contemporary distributions are governed by climate, and how their distributions may move over time in response to climate change, requires that we quantify two very different niche dynamics: (i) filling of the potential niche by the realized niche, which provides insights into the extent to which species will respond to direct versus indirect climatic influences, and (ii) filling of the fundamental niche by the potential niche, which provides insights into where suitable climates exist and are available for colonization. These concepts of niche filling are distinct from similar treatments in the literature that quantify the degree of overlap between species' observed ranges and their potential ranges as estimated from correlative niche models [30], [31]. While the latter concept is central to understanding the predictive accuracy of correlative niche models projected under climate change, the present concepts are used as metrics for understanding the extent to which intrinsic physiological and extrinsic abiotic constraints explain species' distributional limits.

**Figure 1:**
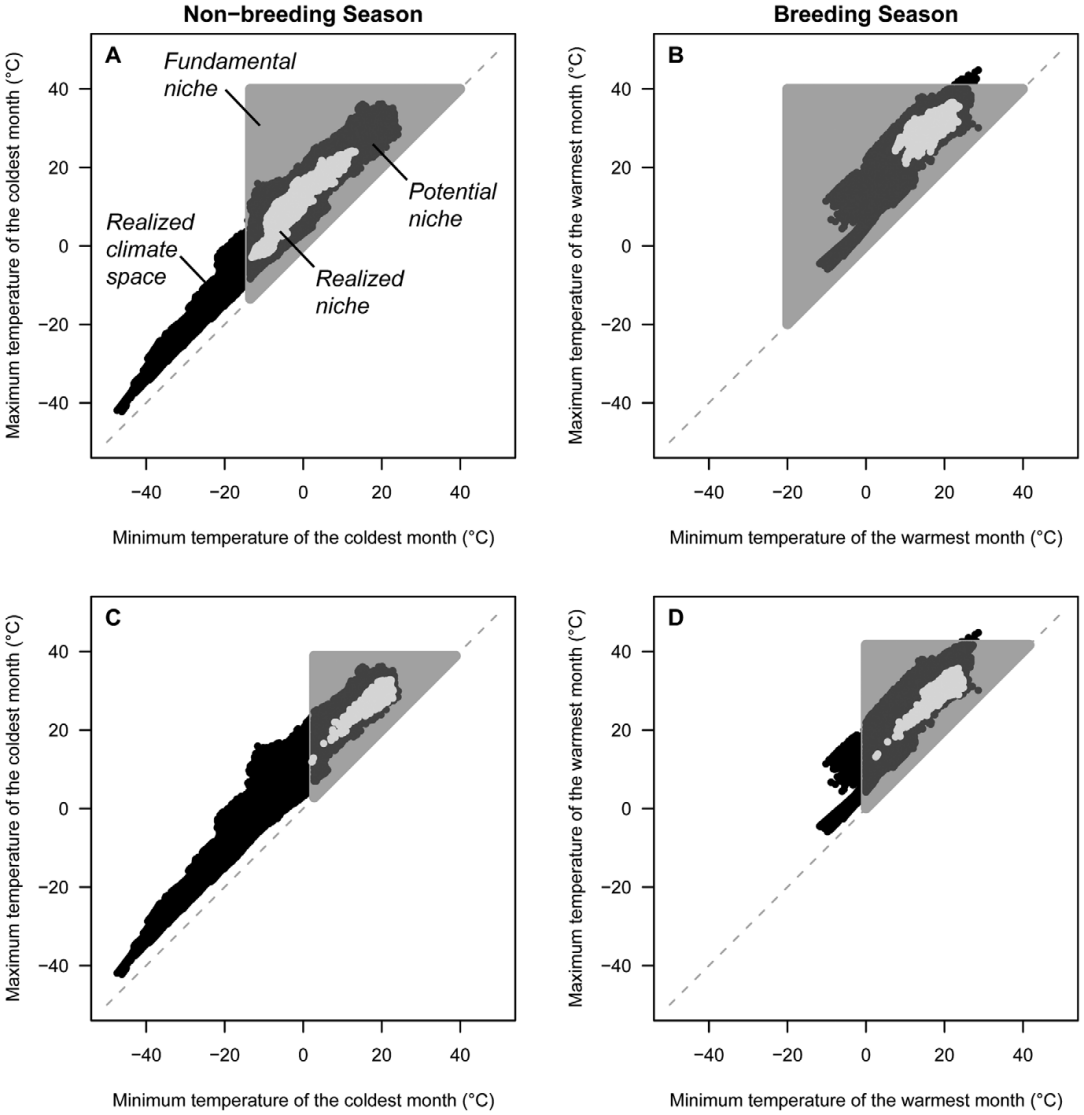
The mechanistic niche model applied to seasonal temperature gradients. One model for the Field Sparrow (A,B) and another for the Variable Seedeater (C,D). Seasonal variation in both the species and the geographic domain is apparent between the non-breeding (A,C) and breeding (B,D) periods. Lower and upper lethal temperatures are used to estimate a fundamental niche (gray triangle), which when intersected with a realized climate space (black points) defines a potential niche (dark gray points) that contains the realized niche (light gray points). The realized climate space is estimated for all of North and South America because the two continents are connected and minimally encompass all of the focal species' distributions.

Temperature gradients are known a priori to influence large-scale distributional dynamics through physiological mechanisms in a variety of taxa [32]–[35]. In an effort to demonstrate the mechanistic niche model in a fashion that is generalized to all species, I focus on major seasonal temperature gradients that relate to individual survival through lethal physiological temperatures. In a simple 2-dimensional environmental space defined by seasonal temperature gradients (Figure 1), a species' fundamental niche is physiologically bounded by its upper and lower lethal temperatures. These temperatures delimit the maximum area of thermal niche space where survival is permissible, although the per capita population growth rate is not necessarily greater than or equal to one. Importantly, not all temperatures exist in the geographic domain at a given point in time, only those defined by the realized climate space, which relates to lower lethal temperature through minimum ambient temperature and to upper lethal temperature through maximum ambient temperature. The potential niche of the organism is defined by the intersection of the fundamental niche with the realized climate space, and within this region exists its realized niche.

When a species' distribution is governed strictly by lethal temperatures, the areas encompassed by its realized (*R*), potential (*O*), and fundamental (*F*) niches are expected to be equal, where *R* = *O* = *F*. However, when other abiotic and biotic factors further shape distribution, as elaborated by Grinnell [15], *R*<*O*<*F*. The ratio of the first two areas, *R*/*O*, describes the proportion of the potential niche actually occupied by the species. When *R*/*O* is less than one, factors such as biotic interactions, barriers to dispersal, and other physiological constraints besides lethal temperature prevent the species from establishing in areas of climate space that are thermally suitable for survival and defined by the geographic domain. Meanwhile, the ratio of the last two areas, *O*/*F*, describes the proportion of each species' fundamental niche that exists in the realized climate space. When *O*/*F* is less than one, physical limits of the realized climate space prevent the species from reaching areas of climate space that are thermally suitable for survival yet undefined by the geographic domain. Hence, by comparing *R*/*O*, *O*/*F*, and their respective deviations from one, it is possible to determine whether a species' realized niche is in physiological equilibrium with the seasonal temperature gradients that shape its potential niche, and whether its fundamental niche as defined by these gradients exists and is available for colonization.

I apply the generalized mechanistic niche model to bird species in North and South America. Owing to seasonal physiological changes or acclimatization in lower and upper lethal temperatures, plus seasonal variation in the realized climate space, I include analyses for both breeding and non-breeding seasons, corresponding to short and long photoperiods of the year.

## Results

The two niche ratios, *R*/*O* and *O*/*F*, exhibited considerable variation across species and seasons (Table 1). While *R*/*O* ranged from 0.02 (Green-backed Sparrow, *Arremonops chloronotus*, breeding and non-breeding) to 0.85 (House Sparrow, *Passer domesticus*, non-breeding), *O*/*F* ranged from 0.08 (Blue-winged Teal, *Anas discors*, breeding) to 0.35 (Yellow-bellied Seedeater, *Sporophila nigricollis*, breeding). The *R*/*O* mean of 0.29 (± 1 SD of 0.24) suggests that focal species are absent from vast areas of the realized climate space that are thermally suitable for survival. Similarly, the *O*/*F* mean of 0.22 (±0.08) indicates that species' lethal temperatures encompass large areas of thermal niche space that do not presently exist in either North or South America.

**Table 1:** Estimates of lower (*T_LL_*) and upper (*T_UL_*) lethal temperatures, and niche area ratios calculated under the mechanistic niche model (*R*/*O*, *O*/*F*), for 12 focal bird species in North and South America.

	Non-breeding season				Breeding season			
Species^a^	*T_LL_* (°C)	*T_UL_* (°C)	*R/O*	*O/F*	*T_LL_* (°C)	*T_UL_* (°C)	*R/O*	*O/F*
Canada Goose	−40.0	41.0	0.28	0.16	−40.0	41.0	0.48	0.10
Blue-winged Teal	−48.0	46.0	0.19	0.12	−42.0	50.0	0.42	0.08
Blue Jay	−30.0	42.5	0.28	0.24	8.0	42.0	0.43	0.32
Blue-black Grassquit	2.8	42.2	0.80	0.22	0.0	43.9	0.30	0.30
Variable Seedeater	2.8	38.9	0.07	0.26	0.0	41.7	0.04	0.33
Yellow-bellied Seedeater	2.8	40.6	0.53	0.24	0.0	40.6	0.22	0.35
Green-backed Sparrow	−8.4	38.9	0.02	0.26	−8.4	41.7	0.02	0.25
American Tree Sparrow	−28.0	47.0	0.25	0.16	−27.6	47.0	0.18	0.12
Field Sparrow	−13.5	40.0	0.18	0.24	−20.0	40.0	0.15	0.18
White-throated Sparrow	−29.0	40.0	0.17	0.20	−29.0	40.0	0.18	0.13
Dickcissel	−4.5	44.0	0.11	0.21	−8.5	44.0	0.14	0.23
House Sparrow	−23.3	42.0	0.85	0.20	−2.5	42.0	0.71	0.30

a Nomenclature follows American Ornithologists' Union [51].

## Discussion

In a simple 2-dimensional environmental space defined by major seasonal temperature gradients, focal bird species possess realized niches that are considerably smaller than their potential niches. They also possess fundamental niches that extend well beyond the realized climate space. Taken in combination, these findings suggest that observed limits on seasonal temperature gradients fail to approximate the species' absolute physiological temperature limits. While it is unknown precisely why the species possess such broad and under-realized physiological tolerances, one explanation is that they evolved throughout pronounced paleoclimate cycles, such as those of the Quaternary [36], that produced combinations of climate that no longer exist today. In light of this possibility, the results also suggest that focal species are physiologically capable of surviving in novel climates that could become available under future climate change; whether colonization occurs will depend on the dispersal capabilities of the organism, the location of the new climates in the geographic domain, and how the multitude of other factors that shape the realized niche change as a consequence of climate change.

Applications of the mechanistic niche model are scale-dependent, so both the results and discussion points below require justification in relation the spatiotemporal scale of the data. Particular explanation is required for the sampling of the realized niche and characterization of the realized climate space. Spatially coarse sampling of the realized niche will lead to errors of commission and an overestimation of its true area. Meanwhile, temporally coarse sampling of the realized climate space will lead to errors of omission and an underestimation of its true area. In practice, this translates to artificially low estimates of *O*/*F* and artificially high estimates of *R*/*O*. Both biases are expected in the present analysis because estimates of *R* derived form coarse range maps [37] and calculations of the realized climate space originated from mean monthly minimum and maximum temperatures averaged over multiple decades [38]. While the bias in *R*/*O* lends further support to the conclusion that focal species are physiologically capable of exploiting unoccupied portions of the realized climate space, the bias in *O*/*F* is potentially problematic because it suggests greater filling of *F* than indicated by the ratios reported Table 1. According to weather data from the National Climate Data Center [39], the record coldest temperature (−66.1°C; North Ice, Greenland; 9 January 1954) is 39% lower and the record warmest temperature (+56.7°C; Death Valley, California, USA; 10 July 1913) is 27% higher than the monthly temperature estimates used to define the realized climate space. Supposing calculations of the realized climate space conservatively underestimated the true value by 50%, all estimates of *O*/*F* reported in Table 1 would still be less than 0.5. Hence, despite measurement uncertainty with *R*/*O* and *O*/*F*, results still suggest that focal species are physiologically capable of colonizing both existing and undefined areas of climate space as captured by major seasonal temperature gradients.

The pronounced levels of seasonal and interspecific variation in *R*/*O* and *O*/*F* are noteworthy in the context of species' tendencies to respond idiosyncratically to climate change [18], [40]. Among focal bird species, seasonal differences in *R*/*O* and *O*/*F* were largely attributed to changes in the size, shape, and position of the realized climate space, which led to dramatic differences in the potential niche between the breeding and non-breeding periods (Figure 1). However, at least for certain species (e.g., Blue Jay, *Cyanocitta cristata*; House Sparrow, *Passer domesticus*), seasonal differences in lower lethal temperature also had a dramatic effect on the potential niche. While upper lethal temperatures were relatively conserved across focal species (+38.9 to 50.0°C), lower lethal temperature ranged from −48.0 to +8.0°C. Most interspecific variation in *O*/*F* could thus be attributed to differences in lower lethal temperature. Unfortunately, interspecific variation in *R*/*O* was not so clear, presumably because species' realized niches were sensitive to different combinations of non-modeled factors. The one interesting observation in this context was that the House Sparrow – an introduced and now naturalized species in North and South America – exhibited the largest estimates of *R*/*O*. This could be explained by a relaxation of the biotic constraints that affect the species throughout its introduced as opposed to native European range, thus enabling greater filling of its potential niche in North and South America. Taken in combination, these observations suggest that – beyond dispersal considerations – species may be responding idiosyncratically to climate change because they vary with regard to the seasons that are most limiting, physiological limits that shape their potential niche, and suite of non-physiological factors that influence their realized niche.

Owing to the challenges of collecting physiological data for multiple parameters, the mechanistic niche model is not primarily intended for use in forecasting, but rather provides a framework for understanding how species respond to particular climatic gradients. Nevertheless, results from the model can be used to improve parameterization of correlative niche models that are projected to the future. For example, the Blue-winged Teal (*Anas discors*) is a remarkable species that is capable of surviving ambient temperatures as low as −48.0°C and high as +50.0°C. Such broad physiological tolerances lead to low values for *O*/*F* (≤0.12) and potential problems with projecting a correlative niche model (trained on present-day observed climate associations) into novel combinations of temperature that are expected to become available with future climate change [16]; questions remain as to whether and how correlative niche models should be extrapolated into new environments [11], [12], [41], [42]. In this case, rather than infer species' temperature limits from an observed contemporary distribution, the correlative niche model might accommodate novel climates by using lower and upper lethal temperatures to establish new niche limits on minimum and maximum temperatures of the coldest and warmest months. As another example, the Green-backed Sparrow (*Arremonops chloronotus*) exhibits such low values for *R*/*O* (0.02) that minimum and maximum temperatures of the coldest and warmest months could be considered biologically irrelevant for the model. Conversely, the introduced House Sparrow (*Passer domesticus*) possesses such high values for *R*/*O* (≥0.71) that even a simple two-variable correlative model would closely approximate both the realized and potential niches. Hence, rather than inferring species' limits from observed climatic associations, physiological data may be used to reparameterize key variables in correlative niche models [42].

Species' distributions are often shaped by different gradients in different areas [15], [43]. The mechanistic niche model is able to identify particular parts of a distribution where lower and upper lethal temperatures, minimum and maximum temperatures of the coldest and warmest periods, and physical limits of the realized climate space constitute limiting factors. For example, during the non-breeding season, lower lethal temperature and minimum temperature of the coldest month prevent the Field Sparrow (*Spizella pusilla*) from moving further polewards in North America (Figure 1A) [9] and limit distribution of the Variable Seedeater (*Sporophila americana*) in cold environments (Figure 1C). Similarly, physical limits of the realized climate space prevent the Variable Seedeater from occupying warmer areas of its fundamental niche (Figure 1C). As temperatures increase during the 21^st^ century, thus generating novel combinations that are presently undefined, the Variable Seedeater could conceivably be expected to colonize existing areas of its fundamental niche that become incorporated into its potential niche – assuming individuals are able to disperse into and establish in areas of the geographic domain that contain the new climates.

Species are also anticipated to respond differently to climate change throughout different parts of their range [44]. As exemplified in Figure 1, species' distributions tend to be limited by physiology at low temperatures and high latitudes or elevations [33], [34]. At high temperatures and low latitudes or elevations, they are often limited by competitive interactions with other species [43]. While notable exceptions to these generalizations certainly exist [45], climate change is hypothesized to affect a large number of species in two primary ways. Throughout cold areas of a distribution, species' responses to climate change are enabled by either a relaxation (warming) or intensification (cooling) of physiological temperature stressors. Meanwhile, throughout warm areas of a distribution, species' responses are mediated by biotic interactions with species in the community. Both types of response are heavily influenced by dispersal. When the rate of temperature change exceeds the rate of dispersal in a geographic domain, or when dispersal rates are characterized by pronounced interspecific variation, species will be in non-equilibrium with respect to the climatic gradients that exert either direct or indirect influences on their realized niches.

One challenge with using the mechanistic niche model to study distributional dynamics lies in defining the geographic domain, which can have a considerable effect on the realized climate space and, by extension, *R*/*O* and *O*/*F*. However, this challenge is not peculiar to the mechanistic niche model. Correlative niche models that rely on the use of pseudoabsence data are also sensitive to the size and shape of the geographic domain [46], as are null biogeographic models [47]. Furthermore, true absence data are difficult to obtain [48] and similarly fail to clarify whether intrinsic (e.g., physiological) or extrinsic (e.g., physical barriers) limits prevent the species from existing in a given area of the geographic domain. In the present study aimed at illustrating the utility of the mechanistic niche model, I calculated the realized climate space based on North and South America because the two continents are connected and minimally encompass all focal bird species' distributions. In general, choosing an appropriately sized geographic domain will depend on a variety of factors, including the timescale of analysis, dispersal capabilities of the organism, and whether analytical constraints necessitate that all focal species share a common domain.

Another challenge with using the mechanistic niche model lies in obtaining the relevant physiological data. Determination of lower and upper lethal temperatures by experimentation is difficult and not even permissible for many species. However, it is important to reiterate that lethal temperatures establish the extralimital bounds of the fundamental niche. Within these bounds, the fundamental niche is a fitness surface that describes the relationship between ambient temperature and other traits that contribute to survival, reproduction, and growth of the population. Hence, lethal temperatures could effectively be replaced by other temperature dependent fitness contours that are measured using standard methods [49]. Examples of such contours in endotherms include lower and upper critical temperatures, which establish the lower and upper bounds of the thermoneutral zone. Such modifications may even be preferable in cases where it is known a priori that lethal temperatures encompass unusually large and physically isolated population sinks, or when it is imperative that the fundamental niche reflect a per capita growth rate of a population as being greater than or equal to one [20], [21]. However, it is useful to reiterate that these marginal populations that lie beyond a traditionally defined fundamental niche are important in an evolutionary context, and their omission may limit our ability to understand how species respond to temperature change.

One major advantage of the mechanistic niche model as detailed here with lethal temperatures is that it is generalized to all species. A downside is that such generality comes at the cost of reality [2], [50], as noted by the low estimates for *R*/*O* in most focal bird species. Importantly, reality in the model may be recovered through inclusion of other relevant variables, and the above comparisons for *R*/*O* and *O*/*F* are also applicable to niche volumes and hypervolumes. However, a distinct challenge lies in parameterizing fundamental and realized niches of high dimensionality and mapping their relation to the realized climate space. In the absence of such detailed knowledge, the mechanistic niche model provides a simple yet robust framework for measuring how intrinsic temperature limits constrain distribution. New applications of the framework stand to greatly inform our understanding of the mechanisms that enable species to respond geographically to climate change.

## Materials and Methods

### Physiological data

Following an extensive literature survey, I selected for analysis 12 bird species with published estimates of lower and upper lethal temperature (Table 1, nomenclature follows American Ornithologists' Union [51]): Canada Goose, *Branta canadensis* [52]; Blue-winged Teal, *Anas discors* [53]; Blue Jay, *Cyanocitta cristata* [54]; Blue-black Grassquit, *Volatinia jacarina* [55]; Variable Seedeater, *Sporophila americana* [55]; Yellow-bellied Seedeater, *Sporophila nigricollis* [55]; Green-backed Sparrow, *Arremonops chloronotus* [55]; American Tree Sparrow, *Spizella arborea* [56]; Field Sparrow, *Spizella pusilla* [57]; White-throated Sparrow, *Zonotrichia albicollis* [58]; Dickcissel, *Spiza americana* [59]; and House Sparrow, *Passer domesticus* [60], [61]. All species are native to North and/or South America, except the House Sparrow, which was introduced from Europe in the mid to late 19^th^ century [62]. Physiological parameters were taken from experimental studies where lethality was defined in terms of 50% mortality on sample populations of acclimated birds. Lethal temperatures were obtained while incrementally changing ambient temperature over a period of multiple days, often lasting weeks, until mortality was reached. Hence, measurements of lethal temperatures were intended to provide a simplified approximation of the fundamental niche [19], [21]. Importantly, the goal of the present study was not to model the complete *n*-dimensional fundamental niche per se, but rather to develop a null model of the fundamental niche for use in determining species' responses to seasonal temperature gradients.

### Temperature data

Temperature data gridded at 10 arc minute spatial resolution for all of North and South America were obtained from WorldClim [38]. I used the original monthly means for minimum and maximum temperature to derive estimates of minimum and maximum temperatures of both the coldest and warmest months. Temperatures of the coldest month were used to define the realized climate space for the non-breeding season and temporally associated with physiological data collected during the short photoperiod, while those from the warmest month were used to calculate the realized climate space for the breeding season and temporally associated with physiological data collected during the long photoperiod. Monthly temperatures provided the best available temporal match to the physiological data, which as described above reflect partial mortality in a population during a significant portion of a month in which a species is exposed to extreme cold or heat stress. However, it is important to note that the temperature estimates derived from means of the daily minimum and maximum temperatures over each month that were further averaged over 50 years. This temporal averaging tended to underestimate the area of the realized climate space with respect to the area of the fundamental niche set by lethal physiological temperature limits (i.e., underestimate the true area of the potential niche); as elaborated in the discussion, the conclusions drawn from the mechanistic niche model are not especially sensitive to this particular bias. Additionally, because of the temporal averaging, it is important to emphasize that results drawn from the model are not necessarily representative of a given year, but rather reflect long-term constraints on contemporary distributions over the past several decades.

### Distribution data

Data on the geographic distributions of focal species were obtained from NatureServe [37]. I converted the vector distribution maps to arrays using the same grid resolution and cell registry as the temperature data. In the case of the six species with strong migratory tendencies (Canada Goose, *Branta Canadensis*; Blue-winged Teal, *Anas discors*; American Tree Sparrow, *Spizella arborea*; Field Sparrow, *Spizella pusilla*; White-throated Sparrow, *Zonotrichia albicollis*; Dickcissel, *Spiza americana*), I only considered areas within the breeding or non-breeding seasonal distributions. While NatureServe data are regarded as providing coarse estimates of the geographic ranges of species, their use in the present study is limited to providing simple approximations of how focal species are distributed with respect to seasonal temperature gradients. As such they likely tend to overestimate the true (unknown) realized niche as defined by the gradients.

### Mapping niche space

Niche space was defined for each species × season using all combinations of minimum and maximum temperatures of both the coldest and warmest months. I used the lower and upper lethal temperatures to establish the lower and upper bounds of the fundamental niche on each temperature gradient. I then intersected each fundamental niche with the realized climate space extracted from WorldClim to obtain an estimate of the potential niche. Within each potential niche, I used the temperature attributes of the NatureServe range maps to characterize each realized niche. In all cases, niche area (*R*, *O*, *F*) was calculated in °C^2^ using temperature data with a precision of 0.1°C. Area estimates were used to calculate *R*/*O* and *O*/*F*, each on a scale from zero to one, with zero indicating maximum discordance and one maximum concordance between each pair of niche spaces.
